# Birth order and pediatric traumatic brain injury

**DOI:** 10.1038/s41598-022-18742-3

**Published:** 2022-08-24

**Authors:** Mazin Omer, Jussi P. Posti, Mika Gissler, Marko Merikukka, Till Bärnighausen, Michael Lowery Wilson

**Affiliations:** 1 Heidelberg Institute of Global Health (HIGH), University of Heidelberg, Heidelberg, Im Neuenheimer Feld 130/3, 69120 Heidelberg, Germany; 2 Injury Epidemiology and Prevention (IEP) Research Group, Turku Brain Injury Centre, Turku University Hospital and University of Turku, Turku, Finland; 3 Neurocenter, Department of Neurosurgery and Turku Brain Injury Center, Turku University Hospital and University of Turku, Turku, Finland; 4 Department of Knowledge Brokers, National Institute for Health and Welfare (THL), Helsinki, Finland; 5 Academic Primary Health Care Centre, Stockholm, Sweden; 6 Department of Molecular Medicine and Surgery, Karolinska Institute, Stockholm, Sweden; 7 Children, Adolescents and Families, National Institute for Health and Welfare (THL), Oulu, Finland; 8 ITLA Children’s Foundation, Helsinki, Finland

**Keywords:** Paediatric research, Epidemiology

## Abstract

Pediatric traumatic brain injury (TBI) is a significant problem of public health importance worldwide. Large population-based studies on the effect of birth order on health phenomena are exceedingly rare. This study examines the relationship between birth order and risk for pediatric TBI among sibling groups. We performed a retrospective cohort study following 59,469 Finnish newborns from 1987 until age 18 years. Data on first diagnosis of TBI was recorded within the 1987 Finnish Birth Cohort (FBC). Compared with first born siblings, later born siblings had an increased risk of TBI during the follow-up period (hazard ratio [HR] 1.02; 95% confidence interval [CI] 0.91–1.14 for second born, HR 1.09; 95% CI 0.95 1.26 for third born, HR 1.28; 95% CI 1.08–1.53 for fourth or higher). When adjusted for sex and maternal age at child’s birth, HRs (95% CIs) for TBI during the follow-up period were 1.12 (0.99–1.26) for second born, 1.31 (1.12–1.53) for third born and 1.61 (1.33–1.95) for fourth born or higher children, respectively. Within this large register-based population-wide study, order of birth modified risk for pediatric TBI among sibling groups. Taken together, these study findings may serve to stimulate further inquiry into genetic, psychological, or psychosocial factors which underlie differences in risk and depth of effect within and between sibling groups.

## Introduction

### Traumatic brain injuries

A traumatic brain injury (TBI) is defined as an alteration in brain function, or other evidence of brain pathology, caused by an external force^1^. These alterations may have immediate, proximate, and long-term consequences on the health and functional well-being of those affected^2^. In 2014, there were 56,800 TBI-related deaths in the US, including 2,529 deaths among children^3^. Severe TBI has a high mortality rate, estimated at 30–40% in observational studies on unselected populations^4^. Moreover, TBI caused 8.1 million years of life lived with disability (YLDs) globally in 2016^5^.

TBIs occur with greater frequency (incidence: 939 cases per 100,000 people)^6^ than all forms of cancers combined^7^ and are among the costliest health conditions to treat and rehabilitate across the entire care continuum^8,9^. The total direct and indirect costs associated with TBIs in Europe have been estimated to be about EUR 33 billion per year^10^. Falls and road injuries are considered the leading causes of new cases of TBI in most countries^5^. In high-income countries, the TBI diagnoses are on the rise among children^11^. Among the pediatric population, special considerations are warranted in the diagnosis and treatment of TBI. These considerations involve taking into account the child's physiology and increased susceptibility for post-traumatic seizures, each within the context of increasing evidence for long-term consequences for even mild TBIs^12^. These long-term consequences include physiological, cognitive, and behavioral changes^12–14^ such as antisocial behaviors, weaker educational performance as well as distress within family and spousal relationships due to the increased care needs of a family member affected by TBI^15–17^, hence, long-term follow-up is often required to confirm physical and cognitive consequences.

### The influence of birth order on health

Classically, birth order refers to the order in which children are born from one set of male and female parents into a family setting^18^. Early research about birth order and its influence on sibling behavior and health was theorized by Austrian physician Alfred Adler^19^. Adler postulated that one's birth position among siblings influences their psycho-social make-up. While little empirical evidence existed initially to support this claim, later research has contributed to the understanding of some of the underlying aspects of the initial hypotheses as they relate to health outcomes^18^.

Personality, genetic, environmental as well as parental factors seem to play a role in forming the relationship between birth order and health conditions^17,20^. Various physical injuries^21,22^, psychological conditions—such as depression, emotional disorders, attention-deficit disorders, suicide attempts, and even intelligence level and handedness^23–25^, were found to have been associated with sibling birth order^26–28^. Mortality risk for some conditions appears to increase within specific birth order categories, and that pattern is relatively stronger among females^29^.

Pediatric TBIs have a complex socio-environmental etiology. More research is needed to understand the particular risks of pediatric TBI, along with efforts to educate parents and caregivers to prevent TBI in this vulnerable group. Sibling birth order remains a relatively unexplored aspect of childhood which might help to explain some of the statistical variation in risk profiles for TBI. The present study examines the hypothesis that later born children are more prone to TBI in comparison to their earlier born siblings. Furthermore, the incidence of TBI among the different birth categories are examined in the study.

## Methods

### Study population

We performed a retrospective cohort study based on data derived from the 1987 Finnish Birth Cohort (FBC). The FBC is a longitudinal nationwide register that includes a complete census of all children born in 1987 in Finland (n = 60,069). The FBC contains information on health and social circumstances from the perinatal period up to and including early adulthood. The cohort contains information about all live and stillborn infants weighing more than 500 g or those with a gestational age of at least 22 weeks. Detailed information about the cohort may be obtained elsewhere^30,31^. The 1987 FBC has an ethics approval from the Finnish Institute for Health and Welfare (THL) (decision §28/2009) and all relevant permissions from all register-keeping authorities. The legal basis for the processing of personal data is public interest and scientific research (EU General Data Protection Regulation 2016/679 (GDPR), Article 6(1)(e) and Article 9(2)(j); Data Protection Act, sections 4 and 6). Since neither cohort members nor their guardians were contacted, no informed consent was required for this study. Informed consent is waived by the ethics committee **(**THL Finnish Institute for Health and Welfare, research ethical working group). All methods were carried out in accordance with relevant guidelines and regulations.

### Inclusion criteria and definitions

TBI diagnoses have been coded according to the International Statistical Classification of Diseases and Related Health Problems (ICD-9: 1987–1995; ICD-10: 1996–2005) as follows: We operationally defined TBI to include cases of concussion, diffuse injury, focal intraparenchymal lesions (such as contusions and intracerebral hematomas) and convexity hematomas, cranial nerve injuries and crush injuries as a result of trauma. Moreover, also fractures of the calvarium and skull base were included (ICD-9 codes 800-801, 803, 804 except for facial traumas, 850-854 and 950-951) (ICD-10 codes S06.0-S06.9, S02.0, S02.1, S02.7-S02.9, S04.0-S04.9, S07.1, S07.8, S07.9, S09.7–S09.9, T02.0, T04.0, T06.0). Co-morbidities at the time of the TBI diagnosis were identified using ICD-9 and ICD-10 as shown in Annex 1^32–35^. Nine cohort members had a diagnosis of a comorbidity at the time of TBI diagnosis. Cohort members who had been diagnosed with a TBI in a hospital setting or visited outpatient clinics (since 1998) in the region were included in this series. Repeated TBIs were defined as having two or more TBIs per child during the follow-up period. Repeated TBI (reported in 13% of the cohort) among individual children were excluded. Thus, we report a single index TBI diagnosis per cohort member. The study sample included all severities of TBI. Hospital diagnoses of TBIs were all obtained from the 1987 FBC which is based on linked and validated administrative register data^30^.

For this study, we identified the children belonging to each set of parents by utilizing the combined parent birth dates that were registered on the birth certificates of each child in the cohort. We included all children with complete information who were followed up from birth until age 18 years (1987–2005) unless they died or emigrated before the year at entry. The final number of included children in the study was 59,469. To reduce possible bias due to e.g. household disruption due to divorce, we restricted the analysis to intact family units consisting of both biological parents who had never been separated/divorced during the cohort period. This was also done to reduce the probability of introducing factors such as having multiple unpaired mothers with the same birth date, or different children residing concurrently with multiple or non-biological parents. Data are presented in three periods each encompassing 5–6 years (1987–1992; 1993–1998; 1999–2005) in order to allow for comparison of the different time periods and evolution of the phenomena over time. Furthermore, data are also presented for the whole period (1987–2005) to have a broad overview of the study cohort. The main outcome of the study is TBI. Validation studies were conducted to ensure the accuracy of measurement of birth order and the outcome in the study^36,37^.

### Statistical analysis

Because all children entered the observation window at the same time point, in 1987, age at entry reflects each person's birth year and is, together with sex and mother's age at delivery, used as a control variable. The incidence rate of TBI was calculated as the number of TBI cases divided by the person time for the children in each birth order during the different periods until TBI, death, emigration, or end of the follow-up. Statistical significance of birth order differences was analyzed using Pearson's chi-square test. To examine the ratio of the risk over time for TBI risk, we calculated hazard ratios (HR) along with their 95% confidence intervals (CI). All statistical tests were two-tailed and the limit for statistical significance was set at *p* < 0.05. We did model performance assessment before our analyses, and all the requirements were fulfilled. The analyses were performed using RStudio Desktop 1.2.5042 for Microsoft Windows. There were no missing data of the participants for the study.

## Results

Table 1 shows the characteristics of the study population (59,469 children of which 30,435 were male and 29,041 females. From these, 1,614 (2.7%) were diagnosed with TBI––61% (984) of whom were males and 39% (630) females. Thirty-eight percent (613) of the diagnosed TBIs were firstborn children; 35.1% (566) among the second-born; 17.2% (278) third born and 9.7% (157) among the latest born children "fourth or higher". Children with mothers aged 25–29 years had the highest percentage of TBIs 35.1% (567), followed by mothers aged 20–24 years old, 24.3% (393) of the total TBIs in the cohort. Most of the TBIs in the study group (54.9%) were diagnosed between the years 1999–2005, The number of diagnosed cases fell substantially to 28.7% between the years 1993–1998 and 16.4% between 1987 and 1992 as shown in Table 2.

**Table 1:** Characteristics of the first time diagnosed TBI in Finland 1987–2005 in Finnish Birth Cohort with a follow-up of 18 years.

Variable	TBI (n = 1 614)	%	No TBI (n = 57 855)	%	*P*-value	Total (n = 59 469)	%
**Birth order**							
First	613	38.0	22 963	39.7	0.1659	23 576	39.7
Second	566	35.1	20 799	36.0	0.4663	21 365	35.9
Third	278	17.2	9 497	16.4	0.3870	9 775	16.4
Fourth or higher	157	9.7	4 586	7.9	0.0084	4 743	8.0
**Maternal age**							
Less than 20	74	4.6	1 847	3.2	0.0015	1 921	3.2
20–24	393	24.3	11 926	20.6	0.0003	12 319	20.7
25–29	567	35.1	21 206	36.7	0.2101	21 773	36.6
30–34	392	24.3	15 179	26.2	0.0790	15 571	26.2
35 or more	188	11.6	7 697	13.3	0.0574	7 885	13.3
**Sex**							
Male	984	60.9	29 451	50.9	< 0.0001	30 435	51.2
Female	630	39.1	28 411	49.1	< 0.0001	29 041	48.8
**First time diagnosed TBI**							
1987–1992	1614	-	57 855		-	59 469	100

**Table 2:** Hazard ratios for pediatric TBI according to sibling birth order 1987–2005.

	First time diagnosed TBI 1987–1992				
	TBI	Crude		Adjusted*	
Birth order	(n = 264)	HR	95% CI	HR	95% CI
First	82	1.00	–	1.00	–
Second	104	1.40	1.05–1.87	1.49	1.10–2.01
Third	47	1.39	0.97–1.98	1.57	1.07–2.31
Fourth or higher	31	1.88	1.25–2.85	2.24	1.42–3.53

	First time diagnosed TBI 1993–1998				
	TBI	Crude		Adjusted*	
Birth order	(n = 464)	HR	95% CI	HR	95% CI
First	172	1.00	–	1.00	–
Second	168	1.08	0.87–1.33	1.20	0.96–1.50
Third	77	1.08	0.83–1.41	1.30	0.98–1.74
Fourth or higher	47	1.36	0.99–1.88	1.71	1.19–2.44

	First time diagnosed TBI 1999–2005				
	TBI	Crude		Adjusted*	
Birth order	(n = 886)	HR	95% CI	HR	95% CI
First	359	1.00	–	1.00	–
Second	294	0.90	0.77–1.05	1.00	0.85–1.18
Third	154	1.03	0.86–1.25	1.25	1.02–1.54
Fourth or higher	79	1.10	0.86–1.40	1.41	1.08–1.84

	First time diagnosed TBI 1987–2005				
	TBI	Crude		Adjusted*	
Birth order	(n = 1614)	HR	95% CI	HR	95% CI
First	613	1.00	–	1.00	–
Second	566	1.02	0.91–1.14	1.12	0.998–1.26
Third	278	1.09	0.95–1.26	1.31	1.12–1.53
Fourth or higher	157	1.28	1.08–1.53	1.61	1.33–1.95

Within the different periods (Table 2), later born “second or more in birth order” was associated with an increased risk of TBI among sibling groups. Compared with earliest-born children, the crude HRs (95% CIs) for second, third, and latest born children diagnosed with TBI within families were 1.02 (0.91–1.14), 1.09 (0.95–1.26), 1.28 (1.08–1.53) during the period 1987–2005, respectively. This association was still apparent after adjusting for sex and maternal age at the child's birth. Here the adjusted HRs (95% CIs) were 1.12 (0.99–1.26), 1.31 (1.12–1.53), 1.61 (1.33–1.95) for the above-mentioned groups, respectively.

Nevertheless, there was a trend of decrease in this association between birth order and TBIs among siblings within the families as we compare the different time intervals (Table 2); the crude HRs (95% CIs) for the latest born children in comparison to the earliest born were 1.88 (1.25–2.85), 1.36 (0.99–1.88), 1.10 (0.86–1.40) in the years 1987–1992, 1993–1998, 1999–2005, respectively. The cross-tabulation of birth order and TBI among siblings (Table 3) showed a statistically significant difference between the eldest in comparison with their younger siblings in the year interval between 1987 and 1992 (82 TBIs vs 182 TBIs, *p* value = 0.004). Otherwise, in the following years as well as in the whole study period, there were no statistically significant differences between the eldest versus non-eldest child (172 vs 292, *p* value = 0.256), (395 TBIs vs 527 TBIs, *p* value = 0.590), (613 TBIs vs 1,001, *p* value = 0.167) in the years 1993–1998, 1999–2005 and 1987–2005, respectively.

**Table 3:** Cross-tabulation of sibling birth order: eldest versus other birth orders.

	Eldest	Not Eldest
**First time diagnosed TBI 1987–1992a**		
No. TBI (%)	82 (0.3)	182 (0.5)
**First time diagnosed TBI 1993–1998b**		
No. TBI (%)	172 (0.7)	292 (0.8)
**First time diagnosed TBI 1999–2005c**		
No. TBI (%)	359 (1.5)	527 (1.5)
**First time diagnosed TBI 1987–2005d**		
No. TBI (%)	613 (2.6)	1001 (2.8)

a chi-square = 8 157, d.f. = 1, *P* = 0 004.

b chi-square = 1 291, d.f. = 1, *P* = 0 256.

c chi-square = 0 291, d.f. = 1, *P* = 0 590.

d chi-square = 1 909, d.f. = 1, *P* = 0 167.

Being a male was associated with a higher risk of being diagnosed with TBI among the siblings within the family in comparison with a female counterpart within the same birth order (Table 4). The HRs (95% CIs) for second, third, and latest born males/females diagnosed with TBI in comparison to the earliest born child diagnosed with TBI within families were 1.21 (1.04–1.41)/0.97 (0.80–1.18), 1.41 (1.16–1.71)/1.17 (0.91–1.50), and 1.63 (1.27–2.09)/1.53(1.13–2.07) for the whole study period between 1987 and 2005, respectively.

**Table 4:** Hazard of TBI by birth order and sex in Finland, 1987–2005.

	First time diagnosed TBI 1987–1992			
	Male (n = 141)		Female (n = 123)	
Birth order	HR	95% CI	HR	95% CI
First	1.00	–	1.00	–
Second	1.83	1.21–2.76	1.17	0.75–1.82
Third	1.86	1.09–3.17	1.29	0.74–2.25
Fourth or higher	2.44	1.27–4.67	2.02	1.07–3.83

	First time diagnosed TBI 1993–1998			
	Male (n = 282)		Female (n = 182)	
Birth order	HR	95% CI	HR	95% CI
First	1.00	–	1.00	–
Second	1.52	1.14–2.02	0.84	0.59–1.20
Third	1.45	0.99–2.13	1.14	0.73–1.78
Fourth or higher	1.94	1.23–3.07	1.45	0.82–2.58

	First time diagnosed TBI 1999–2005			
	Male (n = 561)		Female (n = 325)	
Birth order	HR	95% CI	HR	95% CI
First	1.00	–	1.00	–
Second	0.98	0.80–1.19	1.05	0.81–1.37
Third	1.31	1.02–1.68	1.16	0.82–1.64
Fourth or higher	1.36	0.97–1.91	1.50	0.97–2.31

	First time diagnosed TBI 1987–2005			
	Male (n = 984)		Female (n = 630)	
Birth order	HR	95% CI	HR	95% CI
First	1.00	–	1.00	–
Second	1.21	1.04–1.41	0.97	0.80–1.18
Third	1.41	1.16–1.71	1.17	0.91–1.50
Fourth or higher	1.63	1.27–2.09	1.53	1.13–2.07

Both sexes are adjusted for the joint effect of sex and birth order, as well as the main effects of sex and maternal age at child’s birth.

The incidence of TBI per 100,000 child-year (95% CIs) in the cohort for the whole study period (1987–2005) was as follows; 143.8 (132.4–155.2) for the earliest born, 146.4 (134.4–158.5) for the second born, 157.2 (138.8–175.8) for the third born, 183.8 (155.0–212.6) for the latest born, respectively (Table 5). However, when we compare the incidence between the two periods (1993–1998 vs 1999–2005), we notice that there is an increase in the incidence of diagnosed TBIs among all the sibling groups in the different birth orders; (64.2 vs 83.6), (69.1 vs 75.4), (69.2 vs 86.4), (87.2 vs 91.5) for the earliest, second, third and latest born siblings, respectively.

**Table 5:** Incidence of TBI across birth order categories, 1987–2005.

Birth order	D	Y	I = D/Y	SE	LCI	UCI
**First time diagnosed TBI 1987–1992**						
First	82	128,629.58	63.748	7.039	49.95	77.55
Second	104	116,656.26	89.150	8.741	72.01	106.3
Third	47	53,306.18	88.169	12.860	62.96	113.4
Fourth or higher	31	88.169	120.205	21.589	77.89	162.5
**First time diagnosed TBI 1993–1998**						
First	172	267,887.3	64.206	4.895	54.61	73.8
Second	168	243,048.96	69.121	5.332	58.66	79.57
Third	77	111,160.12	69.269	7.893	53.79	84.74
Fourth or higher	47	53,838.44	87.298	12.733	62.34	112.3
**First time diagnosed TBI 1999–2005**						
First	359	428,999.55	83.683	4.416	75.02	92.43
Second	294	389,640.84	75.454	4.400	66.82	84.08
Third	154	176,741.52	86.415	6.963	72.76	100.1
Fourth or higher	79	85,391.9	91.527	10.297	71.34	111.7
**First time diagnosed TBI 1987–2005**						
First	613	426,154.12	143.844	5.809	132.4	155.2
Second	566	386,427.58	146.469	6.156	134.4	158.5
Third	278	176,741.52	157.291	9.433	138.8	175.8
Fourth or higher	157	85,391.9	183.858	14.673	155.0	212.6

I = incidence, D = TBI cases, Y = person-years = time from birth until TBI, death, emigration or end of the follow-up, LCI = lower bound of confidence interval(95%CI), UCI = upper bound of confidence interval (95%CI).

## Discussion

Our results provide precedent for understanding the relationship between sibling birth order and risk for pediatric TBI. The study reveals that being later-born is associated with an increased risk of a child being diagnosed with a TBI. These results are consistent with prior evidence documenting that various forms of injury occur with greater frequency among later-born children of multiparous families^21,22^.

The total incidence of TBI in the study period ranged from 143.8 for earliest-born to 183.8 for latest born per 100,000. These are considered being within the international range, in which the worldwide incidence of pediatric TBI ranges from 47 to 280 per 100,000^30^. In the U.S. the incidence is 304, United Kingdom 280, and Australia 486 per 100,000 per person-year^38–40^. However, there was a continuous increase in the incidence of pediatric TBI diagnoses over time and among all birth order categories. A study conducted in northern Finland using a cohort of children and young adults born in 1966, showed a lower incidence of 118/100,000 children^41^. The incidence of TBI diagnoses worldwide is rising, partly due to injuries associated with the increased use of motor vehicles, mainly within the world's emerging economies^42^. An important additive risk factor for road injuries is alcohol consumption. A recent study conducted in Finland revealed that due to increases in the taxation of alcoholic beverages over time, alcohol consumption has decreased—in parallel with a reduction in the incidence rate of fatal TBIs for the entire population^43^. An important reason for the high and increasing incidence of TBI diagnoses in pediatric sub-populations in high-income countries is mainly due to the increasing incidence of falls. In a Global School Health Survey covering 26 countries, falls were considered the leading cause of injury among 13–15-year-old adolescents^44^. Moreover, a study conducted in the U.S. revealed that children and adolescents from 0 to 14 years have disproportionately high rates of TBIs due to falls compared with other age categories^45^.

The majority of children diagnosed with a TBI were males: 61% with a ratio of 1 5:1 (M: F). Male over-representation among injury diagnoses is generally consistent with existing literature, in which the reported average ratio for injury is around 1 8:1 (M: F)^38^. In terms of TBI, the incidence has divergent patterns between the sexes––males having a significantly higher incidence––starting from childhood and extending to age 50–60 years. After 60 years of age, the sex-specific incidence rates in males and females become similar. Collins et al.'s data showed that boys were generally less likely to use protective devices and more likely to be injured deliberately. Additionally, boys appear to be at higher risk of injuries that are intentional^46^. Furthermore, widespread cultural and societal norms often allow for greater tolerance for high-risk behaviors among male children, which includes relaxed behavior related to wearing car seatbelts^46,47^. This is consequential since road collisions are a major source of pediatric TBIs^48,49^. Moreover, our study reveals that the incidence of TBI decreases among siblings as they got older, especially within the higher birth order category. Although there existed an association between being later-born and having a higher risk of pediatric TBI, the difference in the risk of TBI between the various birth order categories is getting overtime, as they grew older, statistically insignificant.

The majority of pediatric TBIs occurred among children with younger mothers aged 20–29 years, taking into account that the highest incidence of pediatric TBI was among earliest born children due to the high number of them compared to their siblings from other birth categories. The reason for the high number of earliest born children is that the birth rate has declined considerably in Finland over time as the total fertility rate has diminished markedly in the 2010s^50^.

This trend of increasing risk of injuries among later-born children is mentioned in different studies in low-, middle- as well as high-income countries in the literature “Annex 2”. Orton et al. revealed in a study conducted in the United Kingdom among preschool children that being a later-born child is a risk factor for fracture injuries^21^. Additionally, long bone fractures among children up to 5 years of age, are associated with being later born, in which among the 4th or older sibling there is a threefold increased risk of fractures in comparison to the first child in the family^22^. In Egypt, Halwa et al. also confirmed this relationship by showing that higher birth order in Egypt is correlated with fracture injuries, with more than half of injured children being either second or third in their birth order in their families^51^. Polly et al. claimed according to their study that the increased risk of injury appears to be restricted to later-born siblings in large families^52^.

There are several mechanisms and suggested theories that could generate a relationship between birth order and health, including differences in biological endowments, early parental investment, and later parental or environmental factors^29^. Several explanations about birth order-related injuries have been postulated in recent years. Personality differences seem to play a role, last-born children are described as being athletic, curious, and more aggressive compared to earliest-born children in the same sibling group and hence potentially more outgoing and rebellious^53^. In contrast, later-born siblings are cautious and more fearful^20^. This difference in personality is supported by genetic and environmental evidence. From a genetic standpoint, Scarr et al. suggested that each child in a family receives a random half of each parents' genes, which by chance average to a common sibling share of about half of their genes, and that explains a part of not just the physical differences, but as well the behavioral differences among siblings^54^. This explains partially the difference in personality among siblings and hence the difference in the risk of TBIs. Environmentally, although children share the same household, their experience is different. This due to different factors including sibling interaction with each other, accidental factors of each child's experiences, family composition, extra-family network sources such as peers, teachers, and TV exposure^16^. Parental factors are as well as important, some researchers have suggested that divided attention from the parents in large families may play a role in the increased risk of injuries among later-born children^17^. Mothers may be more cautious in the home environment in dealing with the earliest born children^55^.

One of the strengths of this study is the large, population-based cohort and the long follow-up period of a geographically representative sample. Data were collected through a national health system, which assures uniform access to health care and hence a homogeneous, standardized data collection process. One of the limitations of the study is that we did not consider multiple births, in which the age ranges are thus similar, exposing the entire sibling group to similar risk profiles as they develop. Furthermore, some data of the cohort members could be missing or not well documented, which may as well influence the results of the study. TBI cases could be as well missed, if the diagnosis was made in an outpatient setting before 1996, as this data is not included in the cohort. Furthermore, socio-economic status and rural place of residence, which could increase the risk of exposure to an injury, could not be examined in the study.There is also an aspect in the inclusion criteria that requires attention. We have included patients with calvarial and skull base fractures. The rationale for this is that possible forces, strong enough to cause a skull fracture may injure the underlying brain or result in intracranial bleeding such as epidural hematoma.

## Conclusion

We conclude that there is an association between traumatic brain injuries and being later born in birth order. Due to the potential for serious short and long term consequences, more nuanced research should be carried out with the aim of clarifying which social- biological- and environmental factors influence risk for TBIs within the birth order context. Additionally, further examination of the implications of the phenomena on health systems and public health policies, especially in the setting of low- and middle-income countries, where family sizes are relatively larger^22,51^, is needed. Public health measures to be taken may include expanding public and professional awareness about TBI risks in general as well as maintaining and expanding access to health care providers. Additionally greater emphasis is needed on primary and secondary prevention-related initiatives to reduce the occurrence TBIs and related sequalae and expand research to promote best practices concerning TBI treatment and care for the affected.

## Data access, responsibility and analysis

MO, MLW, MM and MG had full access to all the data in the study and takes responsibility for the integrity of the data and the accuracy of the data analysis.

## Supplementary Information

Supplementary Information.

## Data Availability

The data that support the findings of this study are available from the “National Institute for Health and Welfare (THL)” but restrictions apply to the availability of these data, which were used under license for the current study, and so are not publicly available. Data are however available from “National Institute for Health and Welfare: info@thl.fi” upon reasonable request.

## References

[1] Menon DK, Schwab K, Wright DW, Maas AI (2010). Position statement: Definition of traumatic brain injury. Arch. Phys. Med. Rehabil..

[2] Forslund MV, Perrin PB, Røe C, Sigurdardottir S, Hellstrøm T, Berntsen SA, Lu J, Arango-Lasprilla JC, Andelic N (2019). Global outcome trajectories up to 10 years after moderate to severe traumatic brain injury. Front. Neurol..

[3] TBI-related deaths. Centers for Disease Control and Prevention, https://www.cdc.gov/traumaticbraininjury/data/tbi-deaths.html. Accessed 15 April 2020, March 2019.

[4] Rosenfeld JV, Maas AI, Bragge P, Cristina Morganti-Kossmann M, Manley GT, Gruen RL (2012). Early management of severe traumatic brain injury. The Lancet.

[5] James SL, Theadom A, Ellenbogen RG, Bannick MS, Montjoy-Venning W, Lucchesi LR, Abbasi N, Abdulkader R, Abraha HN, Adsuar JC (2019). Global, regional, and national burden of traumatic brain injury and spinal cord injury, 1990–2016: A systematic analysis for the global burden of disease study 2016. Lancet Neurol..

[6] Dewan MC, Rattani A, Gupta S, Baticulon RE, Hung YC, Punchak M, Agrawal A, Adeleye AO, Shrime MG, Rubiano AM (2018). Estimating the global incidence of traumatic brain injury. J. Neurosurg..

[7] Worldwide Cancer Statistics, Aug 2019. https://www.cancerresearchuk.org/health-professional/cancer-statistics/worldwide-cancer. Accessed 20 April 2020.

[8] Griesbach GS, Kreber LA, Harrington D, Ashley MJ (2015). Post-acute traumatic brain injury rehabilitation: Effects on outcome measures and life care costs. J. Neurotrauma.

[9] Ponsford JL, Spitz G, Cromarty F, Gifford D, Attwood D (2013). Costs of care after traumatic brain injury. J. Neurotrauma.

[10] Olesen J, Gustavsson A, Svensson M, Wittchen H-U, Jönsson B (2012). The economic cost of brain disorders in Europe. Eur. J. Neurol..

[11] Brazinova A, Rehorcikova V, Taylor MS, Buckova V, Majdan M, Psota M, Peeters W, Feigin V, Theadom A, Holkovic L (2016). Epidemiology of traumatic brain injury in Europe: A living systematic review. J. Neurotrauma.

[12] Lloyd J, Wilson ML, Tenovuo O, Saarijärvi S (2015). Outcomes from mild and moderate traumatic brain injuries among children and adolescents: A systematic review of studies from 2008–2013. Brain Inj..

[13] Timonen M, Miettunen J, Hakko H, Zitting P, Veijola J, von Wendt L, Räsänen P (2002). The association of preceding traumatic brain injury with mental disorders, alcoholism and criminality: The northern Finland 1966 birth cohort study. Psychiatry Res..

[14] Gerrard-Morris A, Taylor HG, Yeates KO, Walz NC, Stancin T, Minich N, Wade SL (2010). Cognitive development after traumatic brain injury in young children. J. Int. Neuropsychol. Soc..

[15] Anderson V, Catroppa C, Morse S, Haritou F, Rosenfeld J (2001). Outcome from mild head injury in young children: A prospective study. J. Clin. Exp. Neuropsychol..

[16] Rowe DC, Plomin R (1981). The importance of nonshared (e1) environmental influences in behavioral development. Dev. Psychol..

[17] Lawson DW, Mace R (2009). Trade-offs in modern parenting: A longitudinal study of sibling competition for parental care. Evol. Hum. Behav..

[18] Elliott BA (1992). Birth order and health: Major issues. Soc. Sci. Med..

[19] Hillman BW (1972). The family constellation: A clue to the behavior of elementary school children. Elem. Sch. Guid. Couns..

[20] Sulloway FJ (1995). Birth order and evolutionary psychology: A meta-analytic overview. Psychol. Inq..

[21] Orton E, Kendrick D, West J, Tata LJ (2012). Independent risk factors for injury in pre-school children: Three population-based nested case-control studies using routine primary care data. PLoS ONE.

[22] Baker R, Orton E, Tata LJ, Kendrick D (2015). Risk factors for long-bone fractures in children up to 5 years of age: A nested case–control study. Arch. Dis. Child..

[23] Carballo JJ, García-Nieto R, Álvarez-García R, Caro-Cañizares I, López-Castromán J, Muñoz-Lorenzo L, de Leon-Martinez V, Baca-García E (2013). Sibship size, birth order, family structure and childhood mental disorders. Soc. Psychiatry Psychiatr. Epidemiol..

[24] Rostila M, Saarela J, Kawachi I (2014). Birth order and suicide in adulthood: Evidence from Swedish population data. Am. J. Epidemiol..

[25] Belmont L, Stein ZA, Susser MW (1975). Comparison of associations of birth order with intelligence test score and height. Nature.

[26] Qualter P, Munn P (2002). The separateness of social and emotional loneliness in childhood. J. Child Psychol. Psychiatry.

[27] Bakan P (1971). Handedness and birth order. Nature.

[28] Sudan M, Kheifets LI, Arah OA, Divan HA, Olsen J (2014). Complexities of sibling analysis when exposures and outcomes change with time and birth order. J. Eposure Sci. Environ. Epidemiol..

[29] Black SE, Devereux PJ, Salvanes KG (2016). Healthy (?), wealthy, and wise: Birth order and adult health. Econ. Hum. Biol..

[30] Paananen R, Gissler M (2012). Cohort profile: The 1987 Finnish birth cohort. Int. J. Epidemiol..

[31] Rantakallio P, Jones P, Moring J, Von Wendt L (1997). Association between central nervous system infections during childhood and adult-onset schizophrenia and other psychoses: A 28-year follow-up. Int. J. Epidemiol..

[32] Newton CR (2018). Global burden of pediatric neurological disorders. Semin. Pediatr. Neurol..

[33] Camfield P, Camfield C (2011). Transition to adult care for children with chronic neurological disorders. Ann. Neurol..

[34] Al Salloum AA, El Mouzan MI, Al Omar AA, Al Herbish AS, Qurashi MM (2011). The prevalence of neurological disorders in Saudi children: A community-based study. J. Child Neurol..

[35] Regier DA, Kuhl EA, Kupfer DJ (2013). The DSM-5: Classification and criteria changes. World Psychiatry.

[36] Gissler M, Teperi J, Hemminki E, Meriläinen J (1995). Data quality after restructuring a national medical registry. Scand. J. Soc. Med..

[37] Sund R (2012). Quality of the Finnish Hospital Discharge Register: A systematic review. Scand. J. Public Health..

[38] Dewan MC, Mummareddy N, Wellons JC, Bonfield CM (2016). Epidemiology of global pediatric traumatic brain injury: Qualitative review. World Neurosurg..

[39] Koepsell TD, Rivara FP, Vavilala MS, Wang J, Temkin N, Jaffe KM, Durbin DR (2011). Incidence and descriptive epidemiologic features of traumatic brain injury in King County, Washington. Pediatrics.

[40] Hawley CA, Ward AB, Long J, Owen DW, Magnay AR (2003). Prevalence of traumatic brain injury amongst children admitted to hospital in one health district: A population-based study. Injury.

[41] Winqvist S, Lehtilahti M, Jokelainen J, Luukinen H, Hillbom M (2007). Traumatic brain injuries in children and young adults: A birth cohort study from northern Finland. Neuroepi-demiology.

[42] Roozenbeek B, Maas AIR, Menon DK (2013). Changing patterns in the epidemiology of traumatic brain injury. Nat. Rev. Neurol..

[43] Posti JP, Sankinen M, Sipilä JO, Ruuskanen JO, Rinne J, Rautava P, Kytö V (2019). Fatal traumatic brain injuries during 13 years of successive alcohol tax increases in Finland—A nationwide population-based registry study. Sci. Rep..

[44] Branche C, Ozanne-Smith J, Oyebite K, Hyder AA (2008). World Report on Child Injury Prevention.

[45] Faul M, Wald MM, Xu L, Coronado VG (2010). Traumatic Brain Injury in the United States; Emergency Department Visits, Hospitalizations, and Deaths, 2002–2006.

[46] Collins NC, Molcho M, Carney P, McEvoy L, Geoghegan L, Phillips JP, Nicholson AJ (2013). Are boys and girls that different? An analysis of traumatic brain injury in children. Emerg. Med. J..

[47] Morrongiello BA, Dawber T (2000). Mothers’ responses to sons and daughters engaging in injury-risk behaviors on a playground: Implications for sex differences in injury rates. J. Exp. Child Psychol..

[48] Pearson J, Jeffrey S, Stone DH (2009). Varying gender pattern of childhood injury mortality over time in Scotland. Arch. Dis. Child..

[49] Koskinen S, Alaranta H (2008). Traumatic brain injury in Finland 1991–2005: A nationwide register study of hospitalized and fatal TBI. Brain Inj..

[50] Nikander, T. *Statistics Finland—Births 2018*, April 2020. [Online; accessed 19 April 2020].

[51] Halawa EF, Barakat A, Rizk HI, Moawad EM (2015). Epidemiology of non-fatal injuries among Egyptian children: A community-based cross-sectional survey. BMC Public Health.

[52] Bijur PE, Golding J, Kurzon M (1988). Childhood accidents, family size and birth order. Soc. Sci. Med..

[53] Sulloway FJ (1996). Born to Rebel: Birth Order, Family Dynamics, and Creative Lives.

[54] Scarr S, Grajek S, Lamb ME, Sutton-Smith B, Lamb ME (1982). Similarities and Differences Among Siblings. Sibling Relationships: Their Nature and Significance Across the Lifespan.

[55] Khanom A, Hill RA, Brophy S, Morgan K, Rapport F, Lyons R (2013). Mothers’ perspectives on the delivery of childhood injury messages: A qualitative study from the growing up in Wales, environments for healthy living study (ehl). BMC Public Health.

