# Supplementary material for: Birth order and pediatric traumatic brain injury
Source: Sci Rep. 2022 Aug 24;12:14451. doi: 10.1038/s41598-022-18742-3 (PMC9402548; doi:10.1038/s41598-022-18742-3)
Supplement: Supplementary file 1 — Supplementary Information. [file 41598_2022_18742_MOESM1_ESM.docx]

**Annex 1:**

Co-morbidities; Psychological and neurological disorders at the time of the diagnosis of TBI):

| **Diagnosis** | **ICD-10** | **ICD-9** |
| --- | --- | --- |
| Behavioral and emotional disorders  with onset usually occurring in  childhood and adolescence | (F90-F98) | (300-316)- 299- 783.6- V69.1- 296 – 293.83 - 297 – 295 |
| Neurotic, stress-related and somatoform disorders | (F40-F48) |  |
| Unspecified mental disorder | F99 |  |
| Disorder of psychological development | F80-F89 |  |
| Eating disorders | F50-F59 |  |
| Schizophrenia, schizotypal and delusional disorder | (F20-F29) |  |
| Intellectual disabilities | (F70-F79) |  |
| Extrapyramidal and movement disorders | (G20-G26) | (350-359)- (215.0-215.9)  333- 728- 343- 344- 438.5 - 742.9 – 225- 228.02 – 237.7 – 192.1 – 225.2 – 742- 345 - 333.2 – 331- V17.1 |
| Episodic and paroxysmal disorders | (G40-G47) |  |
| Nerve, nerve root and plexus disorders | (G50-G59) |  |
| Polyneuropathies and other disorders of the peripheral nervous system | (G60-G64) |  |
| Diseases of myoneural junction and muscle (G70-73) | (G70-G73) |  |
| Cerebral palsy and other paralytic syndromes (G80-83) | (G80-G83) |  |
| Other disorders of the nervous system (G90-99) | (G90-G99) |  |
| Benign neoplasms of brain and other parts of central nervous system | D33 |  |
| Congenital malformations of nervous system | (Q00-Q07) |  |
| Cerebrovascular accident | I64 |  |
| Malignant Neoplasm of Brain | C71 |  |
| Malignant Neoplasm of Spinal cord, cranial nerves and other parts of central nervous system | C72 |  |
| Benign Neoplasm of Meninges | D32 |  |
| Sequelae of Intracranial Injury | T90.5 |  |

**Annex 2:**

Applying the Bradford Hill's criteria in the relationship between birth order and traumatic injuries.

| **Bradford Hill's criteria** |  |
| --- | --- |
| Strength of the Association | The majority of groups studying independently the relationship between birth order and health phenomena have *consistently* found an *association* between birth order and traumatic injuries. however, most of these studies were limited to naturalistic follow-up research. |
| Consistency of the Evidence |  |
| Specificity | The *specificity* criterion is unlikely to be met, since Injuries may be influenced by other factors other than birth order. |
| Temporal Sequence | Evidence for the appropriate *temporal sequence* is limited, as prospective studies are needed here in order to prove the sequence. |
| Biological Gradient | In terms of Biological Gradient, the study shows an association between being later in birth order in sibling group and the health phenomena. |
| Biological rational | There was considerable evidence in support of a *biologic rationale* that is *cohesive* with our current knowledge; Birth order has been found to have an effect in the personality, mood and behavior of the individuals and hence their susceptibility to the health phenomena. |
| Coherence | The remaining criteria can be briefly reviewed as follows. In terms of *coherence* with what is known, there is a no consensus as to the role of Birth order in terms of producing Injuries; however, the findings related to biologic plausibility are coherent with what is known about the personality and behavioral differences between birth order categories. There is little research available to support the *experimental evidence* criterion. |
| Experimental Evidence |  |
